# Paramedic mental health literacy and psychiatric emergencies: A qualitative pilot study in Gauteng, South Africa

**DOI:** 10.4102/hsag.v31i0.3241

**Published:** 2026-05-14

**Authors:** Arshaad Boomgaard, John A. Hunter

**Affiliations:** 1 Department of Psychology, Faculty of Humanities and Social Sciences, The Independent Institute of Education (IIE), Emeris, Johannesburg, South Africa

**Keywords:** emergency medical services, mental health literacy, paramedics, pre-hospital care, psychiatric emergencies, South Africa

## Abstract

**Background:**

Paramedics in South Africa frequently respond to psychiatric emergencies in complex, high-risk pre-hospital environments. Their care is shaped by limited formal training, workplace cultures and varying mental health literacy, alongside legal, ethical and contextual constraints specific to emergency medical services. Despite these demands, little research has examined paramedics’ perceptions, attitudes and experiences of psychiatric emergencies in South Africa.

**Aim:**

To explore paramedics’ perceptions, attitudes and experiences of psychiatric emergencies and how they influence assessment, communication and decision-making in the pre-hospital setting.

**Setting:**

Paramedics working in private and public emergency medical services in Gauteng, South Africa.

**Methods:**

An exploratory qualitative design was employed. Semi-structured interviews were conducted with eight paramedics across Basic, Intermediate and Advanced Life Support levels. Data were analysed using inductive thematic analysis to identify patterns across participants’ accounts.

**Results:**

Five themes were identified. Participants described limited formal training in psychiatric emergency care, resulting in reliance on experiential learning and peer modelling. Communication and de-escalation were viewed as essential but inconsistently supported by training and resources. Workplace humour, stigma and emotional distancing were commonly described as coping responses, with divergent views regarding their impact on patient care. Ethical and legal uncertainty shaped conservative or coercive decision-making around consent, restraint and sedation. Cultural, linguistic and environmental factors complicated assessment and engagement.

**Conclusion:**

Despite commitment to supporting psychiatric patients, significant educational and structural gaps constrained effective care. Enhanced training, reflective supervision and context-sensitive guidance may improve patient experiences and paramedic decision-making.

**Contribution:**

This study contributes to understanding how South African paramedics perceive and manage psychiatric emergencies in pre-hospital settings, highlighting educational, ethical, and contextual challenges influencing care.

## Introduction

Psychiatric emergencies constitute a frequent and complex component of pre-hospital emergency medical care. Paramedics are often the first health professionals to encounter individuals experiencing acute psychological distress and are required to make rapid decisions in unpredictable and high-risk environments (Ford-Jones & Chaufan 2017; Govender et al. 2024). Unlike many medical emergencies, psychiatric presentations frequently involve uncertainty regarding patient capacity, consent, and intervention, creating unique clinical, ethical, and legal challenges (Evans, Geduld & Stassen 2018). Both local and international studies suggest that paramedics often report limited formal preparation for managing psychiatric emergencies, with training typically prioritising physical trauma and acute medical conditions (Mothibi, Jama & Adefuye 2019; Parent et al. 2020). In practice, this has been associated with variability in confidence, reliance on experiential learning, and inconsistent approaches to communication and de-escalation (Ford-Jones & Chaufan 2017). However, existing research on paramedic responses to psychiatric emergencies has largely been conducted in high-income settings (e.g. Ford-Jones & Chaufan 2017; Parent et al. 2020), and relatively little is known about how paramedics in South Africa experience and navigate psychiatric emergencies (Govender et al. 2024; Mothibi et al. 2019). In South Africa, pre-hospital emergency care operates within a resource-constrained healthcare system, with paramedics frequently working in settings characterised by high call volumes, safety concerns, and multi-agency involvement (Govender et al. 2024; Rowland & Adefuye 2022). Psychiatric emergencies may involve additional complexities related to uneven access to healthcare services and structural constraints (Govender et al. 2024). Despite these contextual factors, empirical research examining paramedics’ lived experiences of psychiatric emergency care in South Africa remains limited (Mothibi et al. 2019). Prior studies have drawn on frameworks such as stigma (e.g. Goffman 1963), while constructs such as mental health literacy have been developed to explain knowledge, attitudes, and help-seeking in mental health contexts (e.g. Kutcher, Wei & Coniglio 2016). These perspectives highlight how knowledge, attitudes, and workplace norms may influence decision-making and interactional practices. In exploratory qualitative research, however, theory is most appropriately treated as a sensitising lens rather than as a confirmatory explanatory model (Willig 2012). The present study, therefore, prioritises participants’ accounts of practice and decision-making, using theory only to assist interpretation. Against this backdrop, a qualitative pilot study was designed to generate an empirically grounded account of how paramedics in Gauteng experience, interpret, and manage psychiatric emergencies in the pre-hospital setting, with particular attention to training, communication practices, workplace culture, ethical decision-making, and contextual constraints. The study was not intended to evaluate the effectiveness of paramedic care or assess individual competence, but to inform future research and training initiatives while acknowledging the limits of a small qualitative sample.

The study was guided by the following research questions, which were refined inductively during analysis:

1. *How do South African paramedics describe their preparation and training for managing psychiatric emergencies?*
2. *How do paramedics describe their communication and de-escalation practices during psychiatric emergency calls?*
3. *What workplace attitudes, norms, and coping strategies do paramedics report regarding psychiatric patients?*
4. *How do paramedics experience ethical and legal decision-making in psychiatric emergencies?*
5. *What cultural, social, and contextual factors do paramedics perceive as shaping psychiatric emergency care?*

## Research methods and design

### Study design

An exploratory qualitative design was employed, informed by an interpretivist paradigm that emphasises subjective experience within socially situated practice (Willig 2012). This approach was appropriate given the study’s focus on paramedics’ perceptions, attitudes, and experiences of psychiatric emergencies and how these shape assessment, communication, and decision-making in the pre-hospital setting. Rather than testing predefined hypotheses, the study sought to identify patterns and meanings in participants’ accounts of practice.

### Setting

The study was conducted in Gauteng province, South Africa, the country’s most populous province and a key economic centre, characterised by high population density and socio-economic inequality (Statistics South Africa 2019, 2024). Gauteng contains large metropolitan centres as well as peri-urban areas, contributing to diverse service demands and operational contexts for pre-hospital emergency care providers (Statistics South Africa 2024). South Africa’s emergency medical services operate within a dual public–private healthcare system, as established in national health legislation and policy (National Department of Health 2003). National policy documents identify persistent system-level challenges affecting emergency care, including resource constraints, high service demand, workforce shortages, and uneven capacity (National Department of Health 2017). In the mental health sector, longstanding policy and review documents highlight limited availability of specialised services, substantial variability in access – particularly within publicly funded care – and a reliance on emergency and acute services (National Department of Health 2013, 2023). Participants in this study were employed across both public and private emergency care services and were recruited from urban and peri-urban operational areas.

### Study population and sampling

The study population comprised pre-hospital emergency care practitioners registered with the Health Professions Council of South Africa (HPCSA) and practising within Gauteng. Participants held a range of professional qualifications, including Basic Life Support (BLS), Intermediate Life Support (ILS), and Advanced Life Support (ALS) and Emergency Care Practitioner (ECP). A combination of snowball and purposive sampling was used. Initial recruitment commenced through direct contact with an ALS practitioner, who facilitated introductions to additional participants. As recruitment progressed, purposive elements were introduced to ensure variation in qualification level, years of experience, sector (public and private), and operational role. Practitioners with ALS or higher qualifications were more readily accessible and more likely to participate in interviews, resulting in a moderately ALS-weighted sample. This distribution is reported transparently and should not be interpreted as representative of the broader paramedic workforce. Sampling continued until no substantially new conceptual material emerged during analysis, at which point recruitment was discontinued. Given the exploratory and pilot nature of the study, the aim was to achieve depth of understanding within this cohort rather than formal data saturation.

### Data collection

Data were collected through semi-structured, face-to-face interviews conducted by author 1. Interviews took place in private settings at participants’ workplaces or homes and ranged from approximately 10 min to 41 min, with most interviews lasting over 20 min. Variation in interview length reflected differences in participant experience, role, and level of engagement. Shorter interviews provided focused, experience-based accounts on specific aspects of practice. An interview guide was used to explore participants’ experiences, attitudes, and perceptions regarding psychiatric emergencies, as well as training and preparedness, communication strategies, ethical dilemmas, and contextual influences on care.

### Data analysis

Data were analysed using an inductive thematic analysis approach (Braun & Clarke 2006). Both authors independently read and re-read transcripts to achieve familiarisation. Initial coding was conducted manually, without the use of computer-assisted qualitative data analysis software (CAQDAS). Codes were generated inductively from the data and iteratively refined through comparison across transcripts. Both authors independently coded all transcripts. Coding decisions and emerging themes were discussed collaboratively, and codes were revisited and revised to ensure internal coherence and analytic alignment. This approach is consistent with interpretivist qualitative research traditions that emphasise emergent meaning rather than deductive hypothesis testing (Willig 2012; Woiceshyn & Daellenbach 2018).

### Trustworthiness and reflexivity

Trustworthiness was addressed through attention to credibility, dependability, confirmability, and transferability, in line with qualitative guidance (Korstjens & Moser 2018). Credibility was supported through the use of verbatim quotations and close alignment between participants’ accounts and analytic interpretations. Quoted material is referenced using participant identifiers (P1–P8) and transcript paragraph numbers to enhance auditability; participant characteristics are provided in Table 1. In addition, informal post-analysis feedback conversations were conducted with participants who indicated willingness to comment on whether the findings were representative or complete. These conversations were intended to assess resonance rather than to verify transcripts, and no formal member validation was undertaken.

**TABLE 1:** Participant characteristics.

Participant	Qualification	Sector	Primary work setting	Years of experience	Age (years)	Gender
P1	ALS (ECP) (Master’s)	Private	Emergency response and management	6	29	F
P2	ALS (ECP) (Master’s)	Private	Aviation emergency response	5	27	F
P3	BLS	Private	Emergency response	10+	37	M
P4	ILS	Private	Emergency response	8+	34	M
P5	BLS	Private	Emergency response	8+	30	M
P6	ALS (ECP)	Private	Emergency response	1	22	M
P7	ILS	Private	Emergency response	10	30	M
P8	ALS (ECP) (PhD candidate)	Public	Operational pre-hospital care	9+	37	M

F, female; M, male; ALS, advanced life support; BLS, basic life support; ECP, emergency care practitioner; ILS, intermediate life support.

Dependability and confirmability were enhanced through independent coding by both authors, followed by collaborative discussion to resolve interpretive differences. A structured audit trail was maintained throughout the analytic process to document coding decisions, revisions, and thematic development.

Transferability was addressed through a detailed description of the study context, participant characteristics, and analytic process. Reflexivity was considered throughout the study. Author 1 had indirect exposure to paramedic practice through a family member and had previously heard informal accounts of psychiatric emergency care, which was acknowledged as a potential influence. Author 2 had no prior exposure to paramedic practice. To manage potential bias, both authors independently coded transcripts, and interpretations were discussed collaboratively to ensure that themes were grounded in participants’ accounts rather than preconceptions.

### Ethical considerations

Ethical clearance to conduct this study was obtained from the Independent Institute of Education Research Ethics Committee (No. R.0002161 [REC]). Written informed consent was obtained from all participants. Participation was voluntary, and participants were informed of their right to withdraw from the study without consequence. To ensure confidentiality, identifying information was removed during transcription, and participants were assigned pseudonyms (e.g. P1, P2). All electronic data were stored in a password-protected folder accessible only to the two authors. No hard copy transcripts were produced. Data will be retained securely for 5 years before deletion.

## Results

### Participant characteristics

Table 1 summarises the characteristics of the eight participants. Participants ranged in age from their early 20s to late 30s, with professional experience ranging from 1 year to over a decade. The sample included male and female practitioners, the majority of whom were employed in the private sector, reflecting the accessibility of participants during recruitment. Most were operational emergency responders, with one participant occupying a managerial or specialised operational role. Participants represented a range of experience levels, from relatively newly qualified practitioners to those with over a decade of field experience, providing perspectives across different stages of professional development.

### Overview of themes

**FIGURE 1:**
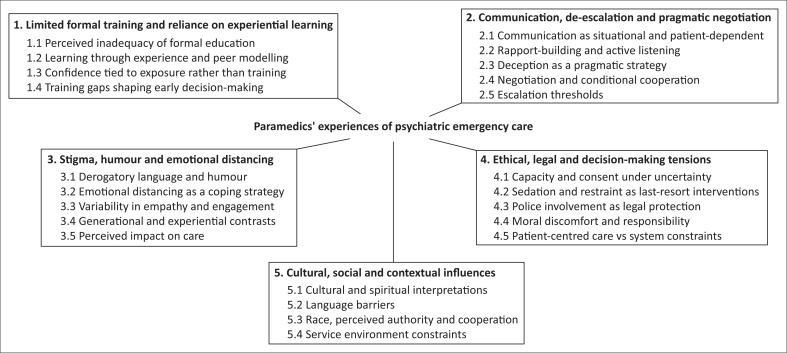
Thematic structure of paramedics’ experiences of psychiatric emergency care, showing five themes and subthemes.

Five themes and associated subthemes were identified across the dataset (Figure 1). These themes reflect paramedics’ accounts of training, communication practices, workplace culture, ethical decision-making, and contextual influences shaping psychiatric emergency care.

#### Theme 1: Limited formal training and reliance on experiential learning

Across interviews, participants described psychiatric emergency care as an area where formal preparation was limited, uneven across qualification levels, and often focused on legal thresholds or sedation rather than communication skills. As a result, participants reported relying on experiential learning, senior guidance, and informal peer modelling to manage psychiatric calls.

**Subtheme 1.1: Perceived inadequacy of formal education:** Participants across BLS, ILS, and ALS (ECP) levels described training in psychiatric emergency care and mental health management as minimal or absent. BLS participants were most explicit about the lack of formal preparation for managing psychiatric emergencies:

‘Well, basically, there is none. The scope there is minimum for basic life support.’ (P3, BLS, M: 008)

‘It’s none. Honestly, it’s not enough.’ (P5, BLS, M: 008)

One participant linked the lack of preparation to the short training duration of training programmes:

‘Their initial course is about 6 or 8 weeks long, so they don’t actually get a lot of time to focus more in depth on mental health illnesses.’ (P1, ALS, F: 007)

Intermediate Life Support training was similarly described as insufficient, with participants indicating limited exposure to psychiatric content:

‘They don’t get taught enough about psychiatric patients.’ (P4, ILS, M: 008)

‘Minimum, unless you are at ALS level. The training is almost minimal.’ (P7, ILS, M: 008)

Advanced life support (ECP) participants also described psychiatric content offered during training as brief and indirect:

‘Very little, it was condensed to one lecture about an hour.’ (P6, ALS, M: 010)

‘It’s very little training and very indirect training.’ (P2, ALS, F: 008)

Psychiatric training within modules was commonly framed as legalistic and sedation-oriented, with limited emphasis on psychosocial approaches such as communication, emotional understanding, and patient-centred engagement:

‘They spoke about the *Mental Health Act* and when we can intervene.’ (P6, ALS, M: 030)

‘We pretty much categorise them into two cases … violent or non-violent.’ (P8, ALS, M: 008)

**Subtheme 1.2: Learning through experience, hierarchy, and peer modelling:** In the absence of structured instruction, participants described learning how to manage psychiatric emergencies through exposure and workplace guidance. BLS participants described following senior staff:

‘Take it from the senior, listen to what they have to say. Or follow the instructions.’ (P3, BLS, M: 010)

Advanced life support (ECP) participants similarly described competence as developing through experience:

‘Once you’re on the road, take it as you go and learn as you go.’ (P2, ALS, F: 008)

‘The more you deal with them … the easier it gets.’ (P1, ALS, F: 044)

Learning was also described as individualised and inconsistent, reflecting the informal nature of workplace-based learning:

‘You kind of develop your own system … Sometimes it works, sometimes it doesn’t.’ (P7, ILS, M: 023)

**Subtheme 1.3: Confidence tied to exposure rather than training:** Preparedness was often portrayed as something that develops over time rather than through education. Senior practitioners described confidence as grounded in experience:

‘I personally feel well prepared … just with the experience that I’ve had.’ (P8, ALS, M: 015)

‘It all comes with experience of your interaction with psych patients.’ (P2, ALS, F: 010)

While expressing confidence based on his experience, P8 acknowledged limited formal knowledge:

‘I know our knowledge is probably quite minimal.’ (P8, ALS, M: 015)

By contrast, BLS and ILS participants more often described uncertainty. One participant stated:

‘You were never well prepared for it.’ (P5, BLS, M: 013)

When asked if he could describe a situation in which he felt well prepared, another participant responded:

‘No.’ (P4, ILS, M: 014)

**Subtheme 1.4: Training gaps shaping early decision-making:** Participants linked limited preparation to narrow response pathways, including early escalation toward transport, South African Police Service (SAPS) involvement, restraint, or sedation. One ALS (ECP) participant described a constrained set of options:

‘The options were either talk to him nicely … or sedate him.’ (P1, ALS, F: 015)

Others described transport as the default response:

‘Our solution to it, majority of the time, is like let’s take the patient and go.’ (P2, ALS, F: 026)

For ALS practitioners, psychiatric callouts were often framed as late-stage escalations for pharmacological intervention rather than initial assessment or de-escalation:

‘Most of the time when I do end up going out to them, it’s for sedation.’ (P6, ALS, M: 016)

While several participants described limited training and reliance on sedation, one participant explicitly linked limitations in training to escalation:

‘If we had better … training … we would more often be able to talk patients down … instead of just using sedation as our end resort.’ (P2, ALS, F: 026)

These accounts suggest that psychiatric emergency care is often approached through experiential and informal learning pathways, with limited formal preparation shaping early decision-making and narrowing perceived response options in the field.

#### Theme 2: Communication, de-escalation, and pragmatic negotiation

Participants consistently described communication as the preferred first-line response in psychiatric emergencies. However, it was primarily framed as a pragmatic tool for reducing risk, securing cooperation, and enabling assessment or transport rather than as therapeutic engagement. When communication was not viable, participants described shifting to negotiation strategies (including strategic framing or deception for some) and, ultimately, crossing escalation thresholds towards SAPS involvement, restraint, or sedation.

**Subtheme 2.1: Communication as situational and patient-dependent:** Participants emphasised that communication was contingent on the patient’s presentation, level of awareness, and risk. Practitioners described quickly assessing patients’ ‘reachability’ and adjusting tone and approach accordingly:

‘Some psych patients just don’t want you to go near them or to treat them at all.’ (P2, ALS, F: 018)

Others stressed that engagement depended on whether the patient could meaningfully respond:

‘Are they stable enough … conscious enough to actually answer our questions correctly …’ (P3, BLS, M: 016)

Communication was also framed as a risk-managed judgement made at the scene:

‘What we usually do … if they are aggressive, we try to talk to them first.’ (P1, ALS, F: 009)

‘It depends on the patient.’ (P3, BLS, M: 026)

**Subtheme 2.2: Rapport-building and active listening:** When communication was possible, participants described rapport-building practices aimed at reducing perceived threat and encouraging cooperation. These practices included speaking calmly, managing body language, and listening carefully to patients’ accounts. These practices included deliberately attempting to engage patients at their level:

‘If you kind of get on their level and talk to them on their level, and you create scenarios that they are comfortable with or conversations that they are comfortable with, it creates a safe environment for them, and they automatically sort of build a bond with you that makes it easier for them to start backing down and cooperate with you.’ (P2, ALS, F: 014)

Participants also described deliberate self-regulation when approaching psychiatric patients:

‘So, we try to go in calmly, in most situations, but we always try to be calm so that our feelings don’t influence them.’ (P1, ALS, F: 011)

Some participants also emphasised the importance of listening to patients:

‘If it seems they need someone to talk to, I’m more than happy to listen … I’m having a conversation with them, mostly to listen to them, not to solve a problem.’ (P8, ALS, M: 017)

Several participants described managing the environment by reducing perceived threat:

‘Taking the medicine … instead of taking a whole bag … it comes over less threatening.’ (P6, ALS, M: 020)

Others emphasised the importance of body language:

‘Meeting someone at eye level rather than towering over them.’ (P8, ALS, M: 047)

Rapport was also framed as instrumental to cooperation:

‘The most important thing is to establish a trust relationship.’ (P1, ALS, F: 013)

**Subtheme 2.3: Deception as a pragmatic de-escalation strategy:** Some participants described deception or strategic reframing as a routine, but uncomfortable, means of securing compliance when direct engagement failed. These accounts ranged from brief references to deception to descriptions of coaching and scenario-building:

‘Lie to them.’ (P4, ILS, M: 020)

‘Sometimes we have to trick them, coach them into the ambulance.’ (P3, BLS, M: 012)

‘You create a scenario … based on what’s going on in their mind … directing it towards what you want.’ (P2, ALS, F: 038)

In some accounts, deception included concealing sedation to avoid force:

‘I can put the medication in the drink, then I don’t have to … tackle them.’ (P1, ALS, F: 048)

Importantly, deception was not universal. P5 did not report deception, and P7 explicitly questioned its usefulness beyond the short-term:

‘Lying doesn’t really work … it can work short-term, but you have to end up at a hospital.’ (P7, ILS, M: 014)

**Subtheme 2.4: Negotiation and conditional cooperation:** Alongside (or instead of) deception, participants described bargaining, conditional reassurance, and prolonged persuasion as means of obtaining cooperation. This was most explicit in ALS (ECP) accounts:

‘You make deals with the patient.’ (P2, ALS, F: 032)

‘Spoke to her for an hour … She eventually agreed to go.’ (P8, ALS, M: 041)

Negotiation was sometimes framed as necessary when sedation was not an option:

‘We knew that sedating her at that point wasn’t an option because in terms of manpower … it was not going to go well. But after lots of talking and lots of convincing … we were able to come to an agreement between her and her family … then she agreed to go with us.’ (P2, ALS, F: 032)

Others described negotiated outcomes shaped by legal limits and autonomy, including refusal pathways:

‘We ended up signing a refusal hospital transfer form … which is his full right.’ (P6, ALS, M: 050)

Consistent with the theme, negotiation was unevenly distributed. It was not clearly described by P3, P4, and P5, whose accounts more often shifted from attempts to calm towards escalation.

**Subtheme 2.5: Escalation thresholds:** Participants described a threshold, typically reached when aggression, refusal accompanied by danger, or other risk markers were present, at which communication and negotiation were abandoned in favour of restraint, SAPS involvement, or sedation. Several participants articulated clear escalation sequences:

‘If that doesn’t help, we try and get SAPS out … we do sedate them.’ (P1, ALS, F: 009)

‘As soon as there’s … aggression … we will intervene very quickly.’ (P6, ALS, M: 026)

For some, paramedic decision-making was described as binary:

‘You’re either sedating, or you’re not.’ (P7, ILS, M: 021)

Overall, these accounts indicate that communication was used less as a therapeutic intervention than as a pragmatic means of managing risk, securing cooperation, and determining when escalation became necessary.

#### Theme 3: Stigma, humour, and emotional distancing in paramedic practice

Participants described workplace talk, humour, and emotional coping responses to psychiatric emergencies, ranging from empathic engagement to stigmatising shorthand and emotional detachment. These practices were largely framed as culturally embedded responses to stress, risk, and repeated exposure within pre-hospital care, rather than expressions of hostility. Importantly, participants varied in their endorsement, resistance, or critical reflection on these practices, with several highlighting potential consequences for patient interactions and the quality of care.

**Subtheme 3.1: Normalised derogatory language and humour:** Participants reported using dismissive labels, shorthand explanations, and humour when referring to psychiatric patients, often described as common workplace discourse rather than deliberate hostility:

‘There were lots of jokes that got made. They would be referred to as “Mike Charlies” … for “mad cow”, or something like that. There were lots of derogatory terms thrown around.’ (P8, ALS, M: 031)

P6 reported the use of common phrases among staff as a recurring feature of workplace culture:

‘Let’s go fight a psych.’ (P6, ALS, M: 038)

P6 explained that such language was often understood among emergency medical service providers as a coping mechanism rather than a literal intention to confront patients:

‘… I think that’s more of a coping mechanism under EMS providers … no one actually goes there with the idea that they want to get into a fight. It’s always we’re there to help the person.’ (P6, ALS, M: 038)

While reflecting on the use of such terms as coping mechanisms, P6 acknowledged that they indicate an underlying stigma associated with psychiatric calls:

‘… I think that’s just one of the stigmatism[s] that’s always there.’ (P6, ALS, M: 038)

Other participants described sceptical shorthand, including assumptions that patients were not genuinely experiencing psychiatric symptoms or that symptoms were attributed to substance use or lifestyle choices:

‘If we know our patient is faking a mental health issue … we don’t always give the most appropriate treatment … because we try to explain that they are wasting resources.’ (P1, ALS, F: 030)

‘We always relate the reason for them having the psych episode based on them having taken something or like a lifestyle choice.’ (P2, ALS, F: 022)

Importantly, this discourse was not uniformly observed or accepted. P3 denied witnessing derogatory language directed at patients and emphasised a boundary between informal joking and professional conduct once on-scene:

‘When we get the call … we actually put our game face on.’ (P3, BLS, M: 024)

**Subtheme 3.2: Emotional distancing as a coping strategy:** Emotional distancing was widely described as a learned coping strategy to manage stress, unpredictability, and safety concerns:

‘But most of the time you just kind of deal with it on-scene and then as soon as we’re done with the patient … we kind of lock that part up or just chat to your colleagues.’ (P1, ALS, F: 025)

‘You can’t be emotional about it all because as soon as you become emotional, mistakes get made.’ (P6, ALS, M: 036)

Others described more generalised detachment, with one participant suggesting that psychiatric calls could lead to emotional disengagement:

‘It’s just one of those calls where you don’t really care.’ (P4, ILS, M: 028)

Several participants distinguished emotional distancing from indifference, emphasising regulation rather than disengagement. One participant stressed the importance of not internalising patients’ behaviour while still recognising the seriousness of the situation:

‘But at the end of the day, you have to understand that even though that patient might be being aggressive or belligerent towards you or nasty towards you, that patient is still in an emergency … you can’t take anything that they do or say personally …’ (P7, ILS, M: 016)

Another participant described emotional distancing as a cumulative coping response shaped by repeated exposure to traumatic events:

‘… part of the ways that we cope with that kind of stuff is to distance ourselves emotionally … and unfortunately, at times dehumanise the situation … I’m not interested in what’s going on with you as a person … I’m just there to stop the bleeding or do a physical job.’ (P8, ALS, M: 051)

P8 also reflected on the risks associated with excessive emotional distancing:

‘That can be part of the coping. But I think sometimes we go too far in that direction.’ (P8, ALS, M: 051)

**Subtheme 3.3: Variability in empathy and engagement:** Participants consistently highlighted variation in how paramedics engage psychiatric patients, both across colleagues and within individual practitioners. Some articulated empathic, person-centred approaches:

‘Treat them as a human … instead of treating them as an object.’ (P3, BLS, M: 050)

‘… come down to their level … and try to understand their background.’ (P5, BLS, M: 011)

Others described colleagues who prioritised task completion or rapid escalation:

‘Let’s just do our job and then leave.’ (P5, BLS, M: 027)

Several ALS practitioners contrasted their own dialogical approaches with coercive practices observed among colleagues:

‘… other people just go in, like listen, you have two options. Either you go willingly, or we’re gonna sedate you.’ (P6, ALS, M: 040)

**Subtheme 3.4: Generational and experiential contrasts:** Differences in engagement were also attributed to experience, exposure, and professional socialisation. Some participants suggested that newly qualified practitioners may be less inclined to spend time listening and may move more quickly toward escalation strategies:

‘… there’s the new guys that are coming out of college, turn 21, don’t really wanna take the time to listen. They just get the police out, sedate, take them to hospital.’ (P5, BLS, M: 029)

Other participants suggested that coercive shortcuts may become normalised among more experienced EMS personnel, particularly when sedation is perceived as the quickest way to resolve complex situations:

‘… I think the older EMS personnel would much rather do that as an easier route because it’s less time-consuming … it’s a matter of getting there, whack them with a whole bunch of sedatives, and then get them in the ambulance.’ (P7, ILS, M: 021)

Participants also linked repeated exposure to traumatic events, fatigue, and cumulative stress to emotional distancing and narrowing professional role conceptions over time:

‘… our jobs can be quite high intensity and high stress … we come across a lot of very difficult things that most people might not ever see or have to deal with in their lives … and we deal with it every month …. ’ (P8, ALS, M: 051)

**Subtheme 3.5: Perceived impact on care:** Participants diverged in their views on whether stigma and emotional distancing influenced patient care. Some minimised its relevance, suggesting that psychiatric patients’ mental state limited the impact of provider attitudes:

‘It don’t impact them really, because they are not in the right sense of mind when treating them.’ (P4, ILS, M: 035)

Other participants explicitly linked these attitudes to reduced assessment depth and a narrowing of care practices:

‘… we fail to kind of investigate other things when we get to the patient … with psych patients we automatically just … get the work done … that compassion sort of falls out with psych patients.’ (P2, ALS, F: 024)

Others reflected on the potential consequences of coercive responses for patient trust and longer-term engagement with services:

‘… you charge in there, you grab this person, you wrestle them to the ground … we provided a very, very negative experience for them … they associate us with a very negative experience …’ (P7, ILS, M: 033)

Participants also raised concerns that forcing psychiatric patients into rigid service pathways could worsen outcomes:

‘… we are going to force you to fit into our system … if you don’t listen to us, I will restrain you … and I will take you to the hospital … and so I think that in the psychological emergency context is quite backwards and will probably cause more harm.’ (P8, ALS, M: 043)

These findings suggest that stigma, humour, and emotional distancing functioned as occupational coping practices, sometimes shaping levels of empathy and engagement in patient interactions.

#### Theme 4: Ethical, legal, and decision-making tensions in psychiatric emergencies

Participants described psychiatric emergencies as ethically complex and procedurally ambiguous, with uncertainty about patient capacity, unclear thresholds for coercion, and competing obligations to protect the patient, the public, and staff. Decision-making was often framed as risk-based and legally constrained rather than therapeutically oriented.

**Subtheme 4.1: Capacity and consent under uncertainty:** Assessing whether a psychiatric patient could refuse care was repeatedly described as difficult, particularly in borderline cases. Several participants described consent as a recurring practical challenge in psychiatric callouts:

‘Challenges? Basically, getting consent for us to treat them.’ (P4, ILS, M: 024)

Participants also emphasised that capacity assessments often relied heavily on individual practitioner judgement in the field:

‘… it’s often left up to you to decide if this person … is compos mentis to make a decision … and that can be a very grey area.’ (P8, ALS, M: 045)

Several participants described psychiatric emergencies as occupying a broader zone of uncertainty within pre-hospital practice, particularly where refusal of care did not clearly meet criteria for coercive intervention:

‘Psych patients in the pre-hospital environment are a very grey area … in terms of how we treat and what we are allowed to do … when sedation becomes illegal … that’s also a grey area.’ (P2, ALS, F: 042)

These situations created ongoing ethical dilemmas about autonomy and implied consent:

‘… am I violating their right to autonomy? Or am I applying the principle of implied consent?’ (P8, ALS, M: 045)

**Subtheme 4.2: Sedation and restraint as last-resort interventions:** Sedation and physical restraint were generally framed as last-resort measures, justified when patients posed a danger to themselves or others. Participants described a stepwise escalation process that began with attempts to communicate and de-escalate situations through conversation:

‘… what we usually do with psych patients, if they are aggressive, we try to talk to them first.’ (P1, ALS, F: 009)

Sedation was described as appropriate primarily when clear risk markers were present:

‘Sedation is normally used when the patients are a threat to themselves or anyone who’s around them.’ (P4, ILS, M: 051)

Some participants highlighted the medical risks associated with pharmacological sedation and therefore expressed a preference for non-pharmacological approaches when possible:

‘… the medications have their own side effects … things like respiratory depression, cardiovascular depression can result.’ (P8, ALS, M: 021)

Despite this reluctance, escalation sometimes felt unavoidable:

‘… we had to make the decision; do we sedate him now or not … I didn’t want to, but it turned out I had to because then he started getting aggressive with us.’ (P8, ALS, M: 041)

When ALS practitioners or pharmacological options were unavailable, physical restraint was positioned as the remaining alternative:

‘If there is no advanced life support … we restrain.’ (P4, ILS, M: 055)

**Subtheme 4.3: Police involvement as legal protection and escalation mechanism:** Police involvement was described as common, particularly when aggression, weapons risk, or refusal of transport raised safety concerns. Some framed SAPS as an escalation step:

‘… if that doesn’t help, we try and get SAPS out …’ (P1, ALS, F: 009)

Others emphasised the role of SAPS as a form of legal protection:

‘… we’ve generally like to have SAPS with us … because legally for us it’s safer …’ (P2, ALS, F: 016)

‘… we mostly see that as … legal protection for us …. ’ (P8, ALS, M: 023)

Participants also noted practical challenges with police involvement, including delays and increased complexity:

‘They sometimes take forever … so sometimes now you sit there with this person suffering for the next four hours, twiddling your thumbs …’ (P8, ALS, M: 023)

‘If they don’t want help … we now need to get this psychiatrist involved, get the police involved … so it becomes a bit challenging in regards to medical care.’ (P5, BLS, M: 019)

In some cases, police presence reportedly increased compliance:

‘… normally when the police show up … they cooperate with us.’ (P4, ILS, M: 067)

**Subtheme 4.4: Moral discomfort and responsibility:** Participants varied in the amount of moral discomfort they expressed. P4, when asked if he could recall any ethical dilemmas, responded:

‘Not really.’ (P4, ILS, M: 061)

Others described ethical dilemmas and competing responsibilities:

‘… there is quite an ethical dilemma … how do you find that balance … what’s the best for the patient … and for the family …. ’ (P2, ALS, F: 036)

Several participants expressed concern about coercion and its longer-term effects:

‘… what negative effect does that have on the patient’s mental health in terms of the long-term care?’ (P7, ILS, M: 033)

P8 also reflected on the ethical cost of coping practices:

‘… part of the ways that we cope with that kind of stuff is to distance ourselves emotionally … and unfortunately, at times dehumanise the situation …. ’ (P8, ALS, M: 051)

**Subtheme 4.5: Patient-centred care and system constraints:** Participants’ accounts revealed a tension between patient-centred ideals and emergency-system constraints, including scope-of-practice limits, training gaps, time pressure, and default transport pathways. Several participants emphasised the importance of building trust and engaging patients through calm communication:

‘… the most important thing is to establish a trust relationship …. ’ (P1, ALS, F: 013)

‘… just treat the person how they want to be treated … never judge them …. ’ (P3, BLS, M: 022)

One participant framed role limits while still emphasising the importance of listening and supportive interaction:

‘… I’m not there to solve your psychological or mental challenges … but … communicating well with you, just being friendly, kind, listening to you, can be helpful …. ’ (P8, ALS, M: 017)

Others described system-driven momentum toward rapid removal from the scene:

‘… our solution to it, majority of the time, is like let’s take the patient and go.’ (P2, ALS, F: 026)

This tension was sometimes explicitly framed as system-centred coercion:

‘… we are going to force you to fit into our system.’ (P8, ALS, M: 043)

Together, these accounts suggest that paramedics’ decisions in psychiatric emergencies were shaped by persistent ethical and legal ambiguity, requiring practitioners to balance autonomy, safety, and system constraints in real time.

#### Theme 5: Cultural, social, and contextual factors shaping psychiatric emergency care

Participants described cultural, social, and contextual dynamics that shaped psychiatric emergency encounters, primarily through their effects on communication, trust, cooperation, and on-scene decision-making. These influences were generally framed as practical constraints on assessment and engagement rather than as explanations for the origins of mental illness.

**Subtheme 5.1: Cultural and spiritual interpretations of symptoms:** Several participants described encountering spiritual or culturally grounded interpretations of psychiatric symptoms that complicated communication and acceptance of medical care. P1 reported that some patients or families interpreted symptoms through ancestral or spiritual frameworks:

‘… they believe that it’s either someone put a spell on them, or it’s the ancestors talking through the patient.’ (P1, ALS, F: 036)

P7 similarly noted that, during psychosis, patients and relatives may understand symptoms through traditional spiritual frameworks:

‘… quite often they will lean more towards that, oh, this is a witch doctor type issue … this is an ancestor-related issue …. ’ (P7, ILS, M: 027)

Participants also described cases in which psychiatric symptoms were expressed through religious or spiritual language during episodes of psychosis:

‘They all think they are God.’ (P4, ILS, M: 030)

The same participant also referred more broadly to the role of traditional belief systems in shaping how symptoms were understood:

‘… these traditional people … they have their own spiritual beliefs.’ (P4, ILS, M: 047)

Across accounts, these interpretations were positioned as interactional challenges affecting rapport and cooperation, rather than as causal theories endorsed by participants.

**Subtheme 5.2: Language barriers:** Language differences were described by a single participant as a direct barrier to assessment, particularly when they limited the ability to determine whether a situation represented a psychiatric emergency or another clinical problem:

‘… there’s often language barriers with various communities that we treat. So sometimes it’s difficult to even know, is this a psychological emergency? Just because the communication is not there.’ (P8, ALS, M: 039)

In these situations, uncertainty was described as encouraging more conservative, system-driven choices (e.g. transport) rather than nuanced on-scene assessment.

**Subtheme 5.3: Race, perceived authority, and cooperation:** Some participants described situational experiences in which provider identity (including race) and perceived authority influenced cooperation and communication:

‘And as soon as they saw that there’s a white guy that arrived, listened to me, no problem, went into the ambulance and left.’ (P8, ALS, M: 039)

P5 similarly described perceived racial preferences in communication, shaped by work in ‘white dominant areas’, noting that patients ‘would rather speak to the white person’, or alternatively might speak to him ‘because I’m Indian’, depending on team composition:

‘… they would rather speak to the white person … or if I’m working with a black person, they would speak to me because I’m Indian.’ (P5, BLS, M: 021)

Additionally, perceived authority was described as affecting compliance, with police presence sometimes increasing cooperation:

‘Yeah, normally when the police show up … they cooperate with us.’ (P4, ILS, M: 067)

These accounts were presented as context-specific observations rather than broad claims about groups.

**Subtheme 5.4: Service environment constraints:** Participants also highlighted environmental and service constraints that shaped risk management and decision-making during psychiatric emergencies. Several risks were described, including limited staffing, volatile scenes, rapid decision-making under pressure, and delays in hospital handover.

One participant noted that response cars may sometimes be staffed by a single provider, increasing vulnerability during psychiatric calls:

‘On the response car we usually only have one person … sometimes I get dispatched to a psych patient, and I get there, and I’m by myself, which obviously puts me more at risk.’ (P1, ALS, F: 038)

Participants also described safety risks associated with volatile or unpredictable environments. P8 described managing a high-risk situation on a highway where provider safety shaped on-scene decisions:

‘… we had to be watching for our own safety with cars flying by.’ (P8, ALS, M: 041)

Alongside these environmental risks, clinical decisions sometimes had to be made under pressure:

‘… we had to make the decision; do we sedate him now or not?’ (P8, ALS, M: 041)

Others described prolonged on-scene waiting in under-resourced or poorly coordinated contexts. For example, one participant described delays when attempting to hand over a psychiatric patient at the hospital:

‘… trying to hand over a psychiatric patient at the government hospital gets a little bit complicated because they don’t want to accept the patient … we must sit with this patient for a lot longer than necessary. And that obviously puts us at risk because they could flip their switch and get pretty angry in a short period of time.’ (P1, ALS, F: 034)

These accounts indicate that psychiatric emergency care was shaped not only by clinical judgement, but also by cultural, linguistic, social, and environmental conditions that influenced communication and operational decision-making.

## Discussion

This study explored paramedics’ perceptions, attitudes, and experiences of psychiatric emergencies and how these factors influence assessment, communication, and decision-making in the pre-hospital setting. The findings suggest a practice environment characterised by limited formal training, reliance on experiential knowledge, and the need to make rapid decisions amid uncertainty, risk, and constrained resources. Similar tensions between emergency care demands and the complexities of psychiatric presentations have been discussed in international research and commentary on pre-hospital mental health care (Ford-Jones & Chaufan 2017; Parent et al. 2020). Across themes, participants described tensions between professional ideals of patient-centred care and the operational realities of emergency work, which together shaped mental health literacy, communication practices, and ethical decision-making.

### Training, mental health literacy, and experiential competence

Consistent with international research, participants described psychiatric training as minimal, fragmented, or peripheral within formal emergency care education (Ford-Jones & Chaufan 2017; Parent et al. 2020). This pattern was evident across qualification levels, though most pronounced among BLS and ILS practitioners. Rather than structured preparation, competence was described as emerging primarily through exposure, repetition, and peer modelling, reflecting the limited and inconsistently integrated mental health education described in the paramedic training literature (Parent et al. 2020).

While experiential learning was associated with increased confidence over time, reliance on informal learning pathways may contribute to uneven skill development and inconsistent approaches to psychiatric care. From a mental health literacy perspective, this pattern reflects a predominantly operational understanding of psychiatric crises (Kutcher et al. 2016). Similar patterns have been reported in pre-hospital mental health research in which behavioural risk and scene safety are prioritised over psychosocial assessment (Mothibi et al. 2019). Early decision-making was therefore often constrained to a narrow set of response options aligned with what practitioners felt equipped to manage in the field.

### Communication and pragmatic de-escalation under constraint

Participants consistently identified communication as the preferred initial strategy when managing psychiatric emergencies, consistent with crisis-management literature emphasising communication and engagement strategies (Evans et al. 2018). However, communication was framed primarily as a pragmatic tool for maintaining safety and enabling transport rather than as a therapeutic intervention. Rapport-building, tone modulation, and body positioning were described as situational techniques, employed when patients were perceived as reachable and risk manageable.

When verbal engagement failed, participants described escalating toward negotiation, strategic framing, or deception. These practices may reflect adaptive responses to operational constraints commonly described in emergency care settings, including time pressure, limited referral options, and uncertainty regarding patient capacity (Evans et al. 2018; Rowland & Adefuye 2022). While such strategies may achieve short-term de-escalation, participants also recognised their ethical ambiguity, particularly where informed consent is compromised. Communication practices were thus shaped less by consistent clinical guidance than by perceived risk thresholds and system demands.

### Stigma, humour, and emotional distancing as coping mechanisms

Workplace humour, derogatory language, and emotional distancing emerged as culturally embedded features of paramedic practice, particularly in relation to psychiatric calls. Participants frequently framed these practices as coping mechanisms rather than expressions of deliberate hostility. Emotional distancing was described as a way to manage cumulative stress, frustration, and exposure to distressing situations, consistent with research documenting emotional strain and attitudinal challenges among emergency workers responding to suicidal patients (Evans et al. 2018).

Participants also acknowledged that such practices could reduce empathy, with implications for quality of care. Importantly, the findings revealed variability in attitudes, with some practitioners resisting stigmatising discourse and emphasising person-centred engagement. This heterogeneity suggests that stigma within pre-hospital care is neither uniform nor inevitable but is embedded in specific cultural and experiential contexts.

### Ethical and legal ambiguity in psychiatric emergencies

Participants described psychiatric emergencies as ethically complex and legally ambiguous, reflecting dilemmas widely reported in emergency mental health care (Evans et al. 2018; Parent et al. 2020). These challenges were particularly evident when assessing patient capacity, consent, and the justification for coercive interventions. Decisions regarding sedation, restraint, and police involvement were framed as last-resort measures, yet participants often felt compelled to act without clear guidance or institutional support.

Police involvement was frequently described as providing legal protection rather than clinical benefit, highlighting the extent to which paramedics operate within overlapping medical and law-enforcement frameworks. Concerns regarding the role of police and the practical challenges of managing suicidal or high-risk patients in the pre-hospital setting have previously been noted in emergency medical services research (Evans et al. 2018). These findings underscore the moral burden placed on practitioners, who must balance patient autonomy with safety obligations, often without access to specialised mental health resources or decision-support tools.

### Cultural and contextual influences on care

Participants emphasised cultural, linguistic, and contextual factors shaping psychiatric emergency care, consistent with research highlighting the influence of social and cultural contexts on understanding and care in mental health (Kutcher et al. 2016). Language barriers, spiritual interpretations of symptoms, and racialised dynamics of trust were described as complicating assessment and communication. These factors were described as practical challenges influencing interaction, cooperation, and decision-making in the field.

Taken together, these findings point to the need for training and policy interventions responsive to the specific social and service contexts in which South African paramedics operate. Enhancing mental health literacy in the pre-hospital setting may therefore require not only expanded education, but also clearer protocols, intersectoral collaboration, and institutional recognition of the ethical complexity inherent in psychiatric emergency care.

### Implications for practice and training

The findings indicate a need for more structured and sustained training in psychiatric emergency care across qualification levels, with emphasis on communication, de-escalation, and ethical decision-making rather than solely on pharmacological or legal thresholds. Similar gaps in mental health preparation for paramedics have been identified in previous research (Mothibi et al. 2019; Parent et al. 2020). The prominence of informal learning and peer modelling in participants’ accounts further highlights the influence of workplace culture, suggesting the potential value of reflective supervision and team-based learning approaches discussed in emergency care training literature (Rowland & Adefuye 2022).

### Limitations and future research

This study was exploratory and based on a small qualitative sample of paramedics working in Gauteng, including those with varying levels of qualification and representing both the private and public sectors of emergency medical services. The findings, therefore, reflect participants’ accounts within a specific operational and geographic context, and readers should interpret them in relation to the particular service environment in which the study was conducted. Future research could extend this work by including a broader representation of public sector services, additional provinces, and perspectives from psychiatric patients and receiving facilities. Quantitative or mixed-methods studies may also help to examine the prevalence and distribution of practices identified in this study. Despite these limitations, the study provides insight into how paramedics experience and navigate psychiatric emergencies in the pre-hospital setting. By foregrounding participants’ accounts, the findings contribute to a growing body of South African research on emergency medical care and identify areas for further inquiry and training development.

## Conclusion

Paramedics demonstrated commitment to supporting psychiatric patients despite significant educational, structural, and contextual challenges. Enhanced psychiatric emergency training, reflective supervision, and improved operational guidance may strengthen patient care and decision-making in South African emergency medical services.
